# A First-Principles Study on the Dislocation Properties of Face-Centered Cubic Metals

**DOI:** 10.3390/ma18030485

**Published:** 2025-01-21

**Authors:** Linghong Liu, Yingqian Han, Touwen Fan

**Affiliations:** 1 School of Electronic Information and Physics, Central South University of Forestry and Technology, Changsha 410004, China; t20060971@csuft.edu.cn; 2 School of Materials Science and Energy Engineering, Central South University of Forestry and Technology, Changsha 410004, China; 3 School of Science, Hunan Institute of Technology, Hengyang 421002, China

**Keywords:** dislocation properties, Peierls–Nabarro model, first-principles calculations, generalized stacking fault energy

## Abstract

The generalized stacking fault energy (GSFE) surface is investigated based on a new anti-alias model and first-principles calculation. Then, based on the outcomes derived from the GSFE surface analysis and the improved Peierls–Nabarro model, the major core properties including core width, Peierls energy, and stress of edge and screw dislocations in Al, Ni, Cu, and Ag are investigated in detail, and the lowest-energy migration pathways for diverse dislocations are investigated. Finally, a quantitative relationship between the ratio of core width to atomic spacing and the Peierls stress is established.

## 1. Introduction

Dislocations, stacking faults, grain boundaries, and other defects play crucial roles in the mechanical properties of crystalline materials [1]. As is well-known, the twinning deformation process initiates with the formation of a twin embryo, which gradually expands through the nucleation and growth of twinning dislocations or dislocation loops. It is universally recognized that twinning dislocations play a pivotal role in the nucleation of twin embryos and the plastic deformation processes of metallic materials. Since the introduction of the concept of dislocation into the field of continuous medium elasticity for the first time in 1907 [2], the role of dislocations as the primary mechanism underlying plastic deformation has gained widespread acceptance. Three independent papers related to dislocations were published simultaneously in 1934 [3,4,5].

Given the crucial role of dislocations in crystal plasticity, researchers have a keen interest in accurately characterizing dislocations at the atomic scale [2,6,7,8,9]. Ungár et al. [7] proposed a dislocation model based on the average square strain to explain the strain anisotropy phenomenon in powder diffraction. The contribution of dislocations to the diffraction patterns of nanocrystals through atomic modeling was analyzed [2]. It was demonstrated that the toughness and ductility of ultra-fine grained Cu could be enhanced, without sacrificing its yield strength, by introducing a high proportion of high-angle grain boundaries and a low density of dislocation [6]. It was found that the previous Volterra dislocation model [2] in elastic continuum was not precise enough, since it does not take into account the nonlinear effect of the dislocation core zone [3]. Currently, there are two prevalent theoretical approaches employed to investigate the properties of dislocation cores. The first involves direct atomistic simulations, relying either on empirical potential energy functions or first-principles calculations [10,11]. However, the reliability of potential functions in precisely delineating the structures of dislocation cores is often questionable, and the calculation outcomes are significantly influenced by the choice of the potential functions. Furthermore, while first-principles calculations provide a high degree of precision in analyzing electronic structures, they come at a relatively high computational cost. Alternatively, the Peierls–Nabarro (P–N) model offers a viable alternative to direct atomistic simulations, serving as a reasonable compromise [12]. Its fundamental concepts can be succinctly summarized in the following three points. Firstly, as the major contribution to the generalized stacking fault energy (GSFE) is from the atoms near the slip surface, the GSFE calculated by using the first- principles supercell method possesses some characteristics of localization. Then, the restoring force can be derived by computing the first-order derivative of energy versus displacement. Finally, applying the restoring force to the P-N dislocation equation greatly improves the P-N theory to obtain more accurate material structure and mechanical properties. In fact, there has been a renewed interest among researchers in the concise P-N model, which was previously proposed for examining the core structures, mobility, and other crucial properties of dislocations [3,4,5]. This method has been extensively applied to study the properties of metals, and their compounds, with dislocations and twins [13].

It is well known that only the intrinsic stacking fault energy (ISFE) can be obtained by experimental measurements, while the other energies on the GSFE curve can only be obtained by atomic simulation. Therefore, the first-principles method is a widely accepted method for calculating GSFE, where the vacuum slab model and alias shear model are two extensively used classic models employed to compute the GSFE of face-centered cubic (FCC) metals [5,11]. Recently, the anti-alias model has been expended to compute the GSFE of crystals with FCC structures [14], which are widely used in hexagonal close-packed (HCP) crystals. It was also proved to have the highest accuracy among the three models.

In the present work, the GSFEs of Al, Cu, Ag, and Ni are calculated using our anti-alias model [14], and different points on the GSFE curve correspond to the stacking fault energies (SFE) with different slip displacements. Then, the P–N model and the GSFE surface are combined to investigate the dislocation core properties and the widths of edge dislocations and screw dislocations; additionally, other structural parameters and mechanical quantities of the four metals are obtained.

## 2. Calculation Method and Theoretical Model

### 2.1. Calculation Method

Up to now, the Vienna ab initio simulation package (VASP) code has been applied widely to calculate total energy [15]. We used the generalized gradient approximation introduced by Perdew, Burke, and Ernzerh (PBE) [16] for the exchange-correlation functional. In order to obtain a more accurate and representative result, we first conducted convergence tests, including K-point, cutoff energy, and the supercell size. The surface of the Brillouin zone was represented by a (23 × 23 × 2) Monkhorst–Pack K-point mesh. A cutoff energy of 350 eV was used for the plane-wave basis set to ensure a good accuracy. The Methfessel–Paxton method (specifically, ISMEAR = 1) was chosen due to its demonstrated suitability for 3D bulk metal systems [17]. The SIGMA parameter determines the width of a smearing; excessively large values may lead to incorrect total energy calculations, while smaller values necessitate a larger K-point mesh. Through meticulous convergence testing, we determined that SIGMA = 0.2 was sufficient for our calculations. As for structural relaxation, all atoms were completely relaxed until the Hellmann–Feynman force on each atom was no more than 0.01 eV/Å, and the convergence criterion of the total energy was 0.01 meV per supercell. When performing calculations to determine the elastic constants, it was crucial to set the key parameter of IBRION = 6. Upon successful completion of the calculation, the elastic stiffness matrix could be obtained from the output file. By leveraging the sophisticated global optimization algorithm of “1stOpt”, we meticulously carried out model validation and parameter identification procedures using the obtained computational data. Consequently, the contour plot of the total dislocation energy surfaces ET could be accurately fitted.

### 2.2. The Generalized Stacking Fault Energy Surfaces

Due to the fact that the closed-packed (111) surface is the gliding surface for FCC metals, the GSFE surfaces of (111) surfaces were computed in this study. The ideal FCC crystal structures had a stacking sequence of ···ABCABC···. We used a supercell with 15 atomic layers along the [111] direction, with 9 atoms per layer, to simulate the shear process. After conducting the necessary tests, we confirmed that the chosen supercell size effectively minimized interactions between stacking faults and satisfied the convergence criteria. This ensures that our calculations accurately represent the 3D bulk properties of the material, providing reliable and reproducible results.

The GSFE surface is defined as the total energy difference in the supercell before and after the relative slip, on the unit area [6]:

(1) $$\gamma (u)=\frac{E\left(u\right)-E\left(0\right)}{A}$$

where E(0) and E(u) represent the total energy of the perfect supercell and the defective supercell, respectively, and A signifies the area of the sliding surface (111). The GSFE surface can be described by a 2D Fourier series with the assistance of a reciprocal lattice [2,14]:

(2) $$\gamma \left(u_{x},u_{y}\right)=c_{0}+c_{1}[\cos\left(2{\mathrm{pu}}_{x}\right)+\cos\left({\mathrm{pu}}_{x}-{\mathrm{qu}}_{y}\right)+\cos\left({\mathrm{pu}}_{x}+{\mathrm{qu}}_{y}\right)\;+c_{2}\left[\cos\left(2{\mathrm{pu}}_{x}-{\mathrm{qu}}_{y}\right)+\cos\left(3{\mathrm{pu}}_{x}-{\mathrm{qu}}_{y}\right)+\cos\left(3{\mathrm{pu}}_{x}+{\mathrm{qu}}_{y}\right)\right]\;+a_{1}\left[\sin\left(2{\mathrm{pu}}_{x}\right)+\sin\left({\mathrm{qu}}_{y}-{\mathrm{pu}}_{x}\right)-\sin\left({\mathrm{pu}}_{x}+{\mathrm{qu}}_{y}\right)\right]$$

where $p=2\pi /\left(\sqrt{3}a_{0}\right),q=2\pi /a_{0}$, a_0_ is the parameter of FCC lattice, and the coefficients c_0_, c_1_, c_2_, and a_1_ can be determined by fitting to the GSFE surface using first-principles calculations. More details can be found in our previous research [14].

## 3. Results and Discussion

### 3.1. Generalized Stacking Fault Energy Surfaces

In this section, a supercell with 15-layer (111) surfaces was applied to calculate the GSFEs of Al, Ag, Cu, and Ni using our anti-alias model. First, the energy and the corresponding coordinate data were recorded when the supercell slipped along the <11$\bar{2}$> and <$\bar{1}$10> directions. Then, we substituted the energy data obtained from slips and corresponding coordinates into Equation (2). Finally, the four coefficients c_0_, c_1_, c_2_, and a_1_ were determined as follows: Al (246.0, −63.0, −17.7, −60.6), Ag (208.7, −53.8, −14.5, 85.1), Cu (329.2, −91.2, −16.8, 144.5), and Ni (479.8, −130.3, −26.1, 177.0) [14].

**Figure 1:**
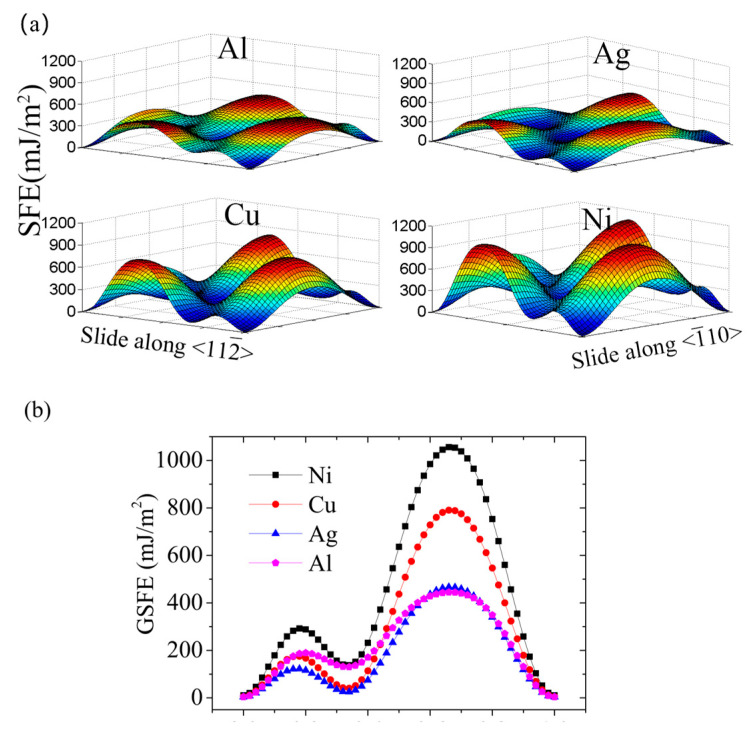
(**a**) The GSFE surface for displacements along (111) plane in Al, Ag, Cu, and Ni. (**b**) GSFE curves of FCC Al, Ag, Cu, and Ni predicted by the periodic supercell method.

Using the fitted parameters, we obtained the GSFE surfaces for the four FCC crystals studied in this research, Al, Ag, Cu and Ni, as depicted in Figure 1a. Obviously, the configurations of these GSFE surfaces are highly similar. The first maximum value encountered along the <11$\bar{2}$> direction corresponds to the unstable stacking fault energy (USFE), representing the lowest energy barrier that must be surpassed for the nucleation of a dislocation. The first minimum value of energy at 1/6<11$\bar{2}$> represents the stable stacking fault (ISF) configuration, from where the dislocation begins to decompose to two Shockley dislocations. However, it is not difficult to note that the projection of the GSFE surface along <$\bar{1}10$> is symmetric, with the point $\sqrt{2}a_{0}/4$ as the center.

Generally, we focused on the stacking faults of FCC crystals in <11$\bar{2}$> direction. It is technically simple to obtain the GSFE curve along <11$\bar{2}$> according to the GSFE surface. Figure 1b indicates that the ISFE and USFE of Al, Ag, Cu, and Ni follow the same order, i.e., γ(Ni) > γ(Al) > γ(Cu) > γ(Ag). However, the ISFE of Ni and Al are far larger than those of Cu and Ag, while the USFE of Cu and Al are relatively close. The specific calculated results are listed in Table 1. It was found that our calculated results of γI and γU for Al, Ag, Cu, Ni are 129.2 mJ/m^2^, 24.6 mJ/m^2^, 40.1 mJ/m^2^, 137.9 mJ/m^2^ and 189.4 mJ/m^2^, 122.3 mJ/m^2^, 175.2 mJ/m^2^, 292.4 mJ/m^2^, respectively. Our results are highly consistent with the reported results.

### 3.2. Dislocation Parameters of Al, Ag, Ni, and Cu

#### 3.2.1. P-N Model

The P-N dislocation model has gained widespread acceptance among researchers and has been extensively utilized to investigate the dislocation properties of metals [25]. It is a very simple method in that only the GSFEs and elastic constants are provided as the basis. In this section, we will further study the dislocation properties of Al, Ag, Ni, and Cu metals based on generalized stacking fault energy.

First, we determined the mechanical parameters for four types of metals by utilizing the stress–strain relationship. For FCC crystals, there are three independent second-order elastic constants, i.e., $C_{11},{\;C}_{12}$, and $C_{44}$. According to the Voigt–Reuss–Hill’s approximation, the bulk modulus B, the shear modulus μ, and the Poisson’s ratio ν are simply obtained.

(3) $$B=(C_{11}+{\;C}_{12})/2,\;\mu =(C_{11}-{\;C}_{12}+3C_{44}/5,\nu \;=\;(3b\;-2\mu )/(6B\;+\;2\mu )$$

Taking advantage of the two-dimensional P-N model, the total energy of dislocation $E_{T}$ mainly includes two parts: elastic strain energy $E_{\mathrm{el}}$ and the atomic dislocation energy of slip surface $E_{A}$:

(4) $$E_{T}(t,d_{ij}(t),\omega_{i}(t),\omega_{j}(t))=E_{el}(d_{ij}(t),\omega_{i}(t),\omega_{j}(t))+E_{A}^{\left(s,e\right)}(t,d_{ij}(t),\omega_{i}(t),\omega_{j}(t))$$

where t (t ∈ (0, 1)) is the position coordinates of the dislocation center, and d and ω represent the separation distance between two partials and the half width of dislocation, respectively, which are obtained by searching for the minimum value point of $E_{T}$.

As for FCC crystals, the pre-logarithmic energy factors Ke and Ks for edge and screw dislocation depend on the elastic constants and dislocation character, and can be calculated from the following formula:

(5) $$K_{e}=(C_{11}+C_{12}){\left[\frac{C_{44}(C_{11}-C_{12})}{C_{11}(C_{11}+C_{12}+2C_{44})}\right]}^{\frac{1}{2}},\;K_{s}={\left[\frac{C_{44}(C_{11}-C_{12})}{2}\right]}^{\frac{1}{2}}$$

As for an isotropic solid, they can be simplified to

(6) $$K_{e}=\frac{\mu }{2\pi (1-\nu )},K_{s}=\frac{\mu }{2\pi }$$

The energy of the dislocation $E_{T}$ changes during slip, and the amplitude of the change is called Peierls energy, which can be obtained by formula (4). Generally, we can achieve the response of a dislocation to an applied stress by minimizing the energy functional related to ρ at a preassigned stress. Then, the critical value of the applied stress that causes this instability is defined as Peierls stress, and it can be obtained through the following formula:

(7) $$P_{F}=-\sum_{n=-\infty }^{\infty }\nabla (\gamma (u((n+t)a^{\prime }))\cdot \rho )$$

where γ, n, and ρ(ρ = du/dη) represent SFE, integer, and dislocation density, respectively, and  a ′ is the periodic constant for the spacing between atom columns.

#### 3.2.2. Dislocation Core Properties

In this part, we focus on the basic properties of the edge dislocation and screw dislocations of Al, Ag, Ni, and Cu, including the separation distance between two partials, Peierls energy and Peierls stress.

Firstly, we can directly extract three independent second-order elastic constants (specifically, $C_{11},{\;C}_{12}$, and $C_{44}$) from the output file generated by the first-principles calculation. Subsequently, by incorporating these constants into formulas (3) and (6), we can derive the shear modulus μ, volume modulus B, Poisson ratio ν, and the dislocation energy factors Ke and Ks. Our calculation results were compared with other reported results in Table 2. The results marked with an asterisk (*) were calculated in this paper using first-principles methods, while the other values are cited from others’ results, with the corresponding references listed thereafter. As we can see, the results are slightly different from one another due to the different computing methods and parameter settings, etc. However, it is reasonable to believe that our results agree sufficiently well with the results listed in the other literature.

The GSFEs of Al, Ag, Ni, and Cu that were obtained in Section 3.1 were employed to substitute the sinusoidal force law within the P-N model. Subsequently, the total energy ET was calculated. By minimizing this total energy ET, the separation distance between two partials of both edge and screw dislocations was determined. The results of these calculations are presented in Table 3. It can be seen that the separation distances between two partials of both edge and screw dislocations of Al are 7.6 Å and 3.5 Å, which are too small to observe in experiment. There is one experimental measurement reported in which the separation distance between two partials of edge dislocation of Al is 8 Å [25], which is in good agreement with our calculation result. Furthermore, the separation distances between two partials of edge dislocations in Ag, Ni, and Cu are 53.7 Å, 47.4 Å, and 19.2 Å, respectively, while the corresponding values of screw dislocations are 21.6 Å, 21.6 Å, and 9.5 Å, respectively. Obviously, the separation distances between two partials of edge dislocations in Al, Ag, Ni, and Cu metals are larger than those of screw dislocations, which indicates that the edge dislocations are much easier to move than screw dislocations.

**Figure 2:**
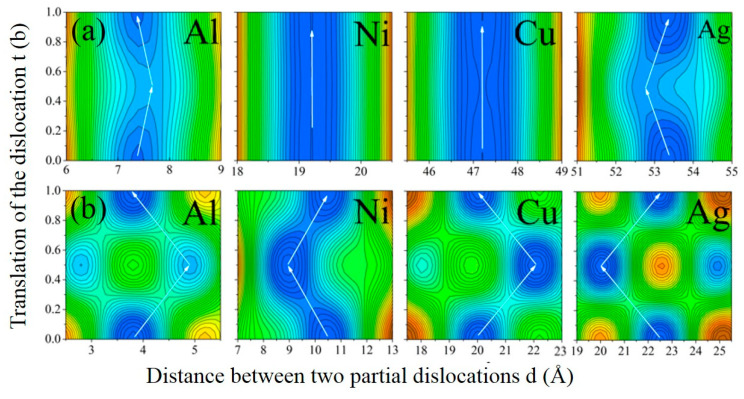
Contour plot of total dislocation energy surfaces ET (t, d) in Al, Ag, Ni, and Cu as a function of dislocation center t and partial separation d: (**a**) edge dislocations; (**b**) screw dislocations.

When a dislocation moves, its configuration and the separation distances between two partials of the dislocations d will change, such as from one dislocation configuration to another one with the lowest dislocation energy. Based on seeking the minimum value of ET using universal global optimization, the functional relationship ET (t, d) was derived. The dislocation energy surface in Figure 2 shows the energy variation with d and t. It was found that the number of minimum local dislocation energies for various dislocations is different. As for screw dislocations, there are several minimum local energies, but only one minimum value for edge dislocations. Obviously, some stable configuration of dislocation with a small local energy will be formed during the process of movement, and the distance d will change when the dislocation moves to a new stable state. From the perspective of energy, dislocations will move along the “pathway” with the lowest energy, and the Peierls energy can be described by the difference to the highest saddle point along the pathway [35,36]. The white arrows denote the lowest energy reaction paths for various dislocations, while the positions with the lowest local energy are highlighted in blue. As the dislocations move, the partial separation ‘d’ of Ni and Cu remains virtually unchanged, suggesting that both partials maintain synchronized motion. However, as the dislocation center ‘t’ increases, the partial separation ‘d’ of Al also rises. As is well-established, Burgers vectors, which delineate lattice distortions induced by dislocations within crystal structures, play a pivotal role in comprehending the mechanical properties of materials. Their magnitude can be computed using theoretical formulas, while experimental techniques such as high-resolution transmission electron microscopy (HRTEM) or X-ray analysis also offer means to ascertain their values [37]. When a dislocation moves a distance equivalent to half a Burgers vector, the partial separation ‘d’ initially decreases, and then subsequently returns to its initial state (t = 0) upon moving a full Burgers vector distance. Conversely, the situation in Ag is the opposite to that in Al. Nevertheless, the variations in ‘d’ for both Al and Ag are minimal and can be disregarded.

Regarding screw dislocation, the situation varies. When the dislocation remains stationary (t = 0), the screw dislocations in Al, Ni, Cu, and Ag exist in a slightly extended stable state, characterized by decomposition distances of 3.82 Å, 10.50 Å, 20.4 Å, and 22.48 Å, respectively. As the moving distance of dislocation increases to t = b/2, the decomposition distances of the extended stable states vary to d = 4.91 Å, 8.94 Å, 22.08 Å, and 20.04 Å, respectively. Finally, the dislocation displacement arrives at t = b, and the dislocation structure is consistent with t = 0 according to the periodicity of the crystal.

In addition to describing the structure of the dislocation core, it is convenient for us to obtain PE and PF through the total energy Equation (4) and the Peierls stress Equation (7) along the reaction path. The geometric parameters of dislocation play a very important role in the calculation of interfacial stress, and the maximum stress is Peierls stress. Our calculation results, along with other published findings, are summarized in Table 4. It can be seen that the Peierls energy and Peierls force of screw dislocation are far greater than that of edge dislocation. It is shown that the edge dislocation is much easier to move than the screw dislocation in FCC crystals, which is consistent with the conclusion of Yasi et al. [38].

## 4. Conclusions

In summary, we initially calculated the generalized stacking fault energy (GSFE) surfaces for Al, Ag, Cu, and Ni using our anti-alias model. Subsequently, we conducted an investigation into the characteristics of edge and screw dislocations in these four materials by incorporating the GSFE surfaces with the two-dimensional Peierls–Nabarro (P-N) model. The findings are as follows:

(1) Both types of dislocations split into two partials, with screw dislocations exhibiting multiple stable low-energy states, whereas edge dislocations possess only a single stable state.
(2) The decomposition width of edge dislocations in Al, Ag, Cu, and Ni crystals is greater than that of screw dislocations. However, the Peierls energy and Peierls force associated with screw dislocations are higher than those of edge dislocations, suggesting that edge dislocations exhibit superior mobility and are therefore easier to move compared to screw dislocations.

Our calculation results offer valuable insights into the mechanical properties, plastic deformation, and microstructure of materials, particularly for Al where the narrow decomposition width of dislocations poses significant experimental challenges. These findings serve as crucial theoretical guidance in material design and performance optimization.

## Figures and Tables

**Table 1:** The ISFE and USFE of Al, Ag, Cu, and Ni (mJ/m^2^), where * represents our calculated results.

	γ_I_	γ_U_
Al	129.2 *	189.4 *
	133 [13], 158 [18], 153 [19], 146 [20], 142 [21], 130 [22], 142.4 [23]	189 [13], 225 [18], 178 [20], 185 [21], 162 [22], 177.4 [23]
Ag	24.6 *	122.3 *
	26 [13], 18 [22]	119 [13], 133 [22]
Cu	40.1 *	175.2 *
	42 [13], 43 [18], 51 [19], 38 [20], 39 [21], 41 [22], 40.5 [23]	171 [13], 175 [18], 164 [20], 177 [21], 180 [22], 161 [23]
Ni	137.9 *	292.4 *
	129 [13], 137 [20], 122 [21], 110 [22], 132 [24]	280 [13], 278 [20], 299 [21], 273 [22], 305 [24]

**Table 2:** Mechanical parameters of Al, Ag, Ni, and Cu, where * represents our calculated results.

	Al	Ag	Cu	Ni
C11	102.2 *	112.3 *	175.9 *	255.7 *
	111 [26],114 [27]	132 [27]	180 [26], 167 [28],	233 [28]
C12	65.2 *	82.7 *	126.4 *	176.4 *
	56 [26],62 [27]	97 [27], 91 [28]	124 [26], 124.9 [23], 124 [28]	154 [28]
C44	27.6 *	34.1 *	72.8 *	114.1 *
	32 [26]	57 [28]	84 [26],76 [28]	128 [28]
μ	23.9 *	26.4 *	53.6 *	84.3 *
	28.8 [29]			
B	77.5 *	92.5 *	142.9 *	202.8 *
		104 [28]	138 [28]	180.4 [28]
ν	0.36 *	0.37 *	0.33 *	0.32 *
	0.344 [29]			
Ke (GPa)	5.9 *	6.7 *	12.8 *	19.7 *
Ks (GPa)	3.8 *	4.2 *	8.5 *	13.4 *

**Table 3:** The separation distance between two partials of edge and screw dislocations in Al, Ag, Ni, and Cu, where * represents our calculated results.

	Edge (Å)	Screw (Å)
Al	7.6 *	3.5 *
	10.3 [18], 7.2 [19], 3.5 [30], 7.4 [31], 5.6 [32], 8.0 [33]	2.1 [30], 4.9 [34]
Ag	53.7 *	21.6 *
Cu	47.4 *	21.6 *
Ni	19.2 *	9.5 *

**Table 4:** The calculated Peierls energy (PE) and Peierls stress (PF) of edge dislocations and screw dislocations in Al, Ni, Cu, and Ag crystals, where * represents our calculated results.

	Edge				Screw			
	Al	Cu	Ag	Ni	Al	Cu	Ag	Ni
PF (MPa)	2.699 *3.2 [30]	0.078 *0.003 [39]	0.684 *0.82 [40]	0.049 *	324.140 *256 [30]	57.959 *	103.142 *	171.888 *
PE (meV/Å)	0.044 *	0.001 *	0.010 *	0.001 *	3.761 *	0.591 *	1.149 *	0.885 *

## Data Availability

The original contributions presented in this study are included in the article. Further inquiries can be directed to the corresponding author.
